# Hygiene and biosecurity protocols reduce infection prevalence but do not improve fledging success in an endangered parrot

**DOI:** 10.1038/s41598-019-41323-w

**Published:** 2019-03-18

**Authors:** Deborah J. Fogell, Jim J. Groombridge, Simon Tollington, Stefano Canessa, Sion Henshaw, Nicolas Zuel, Carl G. Jones, Andrew Greenwood, John G. Ewen

**Affiliations:** 1 Durrell Institute of Conservation and Ecology, School of Anthropology and Conservation, University of Kent, Canterbury, CT2 7NZ UK; 2 Institute of Zoology, Zoological Society of London, Regents Park, London, NW1 4RY UK; 3 North of England Zoological Society, Chester Zoo, Cedar House, Caughall Road, Chester, CH2 1LH UK; 4 Wildlife Health Ghent, Department of Pathology, Bacteriology and Avian Diseases, Faculty of Veterinary Medicine, Ghent University, Merelbeke, Belgium; 5 Mauritian Wildlife Foundation, Grannum Road, Vacoas, Mauritius; 6 Durrell Wildlife Conservation Trust, Les Augres Manor, Trinity, Jersey JE3 5BP UK; 7 International Zoo Veterinary Group, Station House, Parkwood Street Keighley, West Yorkshire, BD21 4NQ UK

## Abstract

Emerging Infectious Diseases (EIDs) are recognised as global extinction drivers of threatened species. Unfortunately, biodiversity managers have few tested solutions to manage them when often the desperate need for solutions necessitates a response. Here we test *in situ* biosecurity protocols to assess the efficacy of managing Psittacine beak and feather disease (PBFD), one of the most common and emergent viral diseases in wild parrots (Psittaciformes) that is currently affecting numerous threatened species globally. In response to an outbreak of PBFD in Mauritius “echo” parakeets (*Psittacula eques*), managers implemented a set of biosecurity protocols to limit transmission and impact of Beak and feather disease virus (BFDV). Here we used a reciprocal design experiment on the wild population to test whether BFDV management reduced viral prevalence and viral load, and improved nestling body condition and fledge success. Whilst management reduced the probability of nestling infection by approximately 11% there was no observed impact on BFDV load and nestling body condition. In contrast to expectations there was lower fledge success in nests with added BFDV biosecurity (83% in untreated vs. 79% in treated nests). Our results clearly illustrate that management for wildlife conservation should be critically evaluated through targeted monitoring and experimental manipulation, and this evaluation should always focus on the fundamental objective of conservation.

## Introduction

Emerging infectious diseases (EIDs) are key contributors to the current global biodiversity crisis^1,2^. While population biologists recognize infectious pathogens as an integral and constant mechanism for evolutionary change within natural populations^3^, the emergence of novel pathogens may increase the risk of extinction for vulnerable species and populations^4^. Viruses are responsible for over 40% of all recently surveyed wildlife EIDs^5,6^, and have thus been highlighted as a particular threat to wildlife. The threats from viruses are in part due to their ability to adapt rapidly to novel hosts^7,8^, conferring the capacity to become infectious across a wide host range^7^.

Conservationists have struggled in the face of EIDs. Broadly speaking, management of EIDs can be broken down into three main types of strategies. First, those that target direct treatment or vaccination of the infected host, such as anti-fungal treatment of amphibians affected by *Batrachochytrium dendrobatidis*^9,10^ or the inoculation of black-footed ferrets against canine distemper virus^11^. Second, strategies that aim to prevent interaction between disease vectors and the focal host, such as pesticide application for reducing tick populations that are responsible for the spread of Lyme disease^12^. Third, strategies that aim to reduce the risk of transmission through hygiene, biosecurity or direct treatment of environmental reservoirs^13^. For example, the disinfection of water bodies associated with the spread of avian cholera^14^ and liming around feeding stations to reduce the prevalence of lungworms in hares^15^. Various combinations of these strategies have been broadly applied across taxonomic groups. In extreme cases these disease management strategies can be combined with the removal of surviving individuals to captivity^16^.

Management actions aimed at reducing EID transmission *in situ* are mostly reactive and the efficacy of only a few have been thoroughly assessed^13,17,18^. These management actions are often modified versions of those used in clinical settings and based on expert knowledge of wildlife health specialists. However, their application is rarely backed by critical evaluation of their ability to reduce transmission (the means to threatened host species recovery) and aid recovery of the threatened host species (the fundamental objective). This raises a dual concern that conservation management may continue despite it being ineffective or even detrimental to endangered species recovery, and that this may add unnecessary financial and logistical burdens to management.

Psittaciformes (parrots) are one of the most vulnerable avian orders, with over a quarter of all extant species recognised as in need of conservation action and 75% of species in population decline^19^. One major threat to parrots has been the emergence and global spread of Psittacine beak and feather disease (PBFD), one of the most common viral diseases in wild parrots^20–22^. PBFD was first described in the mid-1970s, originating in the South Pacific and is spreading rapidly across the world^22–24^. PBFD is caused by the Beak and feather disease virus (BFDV) and the disease has been implicated in the decline of many wild parrot populations, including the endangered Cape parrot (*Poicephalus robustus*) of South Africa^25^, the Australian orange-bellied parrot (*Neophema chrysogaster*)^26^ and the Mauritius “echo” parakeet (*Psittacula eques*)^27^. Concern about the threat of PBFD in Australia has led to it being listed as a “Key Threatening Process” to biodiversity^28^. The emergence of PBFD has directly impacted species recovery programmes by altering how and what management tools are used (e.g. captive breeding, translocation, cross fostering^29,30^).

Despite calls to more directly manage PBFD only a limited range of management actions have been developed, most focussing on hygiene and biosecurity. In Australia, for example, a detailed Threat Abatement Plan for BFDV includes the use of disinfectants in nest and transport boxes^28^. However, the same Threat Abatement Plan also notes that there is no assurance as to whether recommended actions will actually reduce transmission. To our knowledge, there are no studies that provide empirical evidence for the effectiveness of *in situ* biosecurity management actions to reduce BFDV transmission. Research into the efficacy of biosecurity interventions is therefore paramount to improve our ability to carry out evidence-based management of endangered parrot species in the face of BFDV.

In this study, we experimentally test the performance of nest site biosecurity for reducing BFDV transmission and enhancing Mauritius parakeet population recovery. Mauritius parakeets were once the world’s rarest parrot, numbering fewer than 20 individuals in the early 1980s^31^. Intensive management has increased their abundance to 136 breeding pairs in 2017^32^. However, these efforts were interrupted by an outbreak of PBFD in 2005^27^. Unfortunately, management actions for Mauritius parakeets such as cross-fostering offspring between nests, captive rearing and release of chicks between subpopulations, and the aggregation of individuals at supplementary feeding hoppers are thought to increase horizontal BFDV transmission^33^. Consequently, management actions including the movement of eggs and individuals between sites were ceased and additional, rigorous biosecurity was implemented at nest sites. Supplementary feeding, however, has been maintained as it’s demonstrated to improve fecundity^29^. Nest site management comprises three elements: (i) wearing medical barrier suits whilst accessing nests, (ii) disinfecting nest sites with an anti-viral solution and (iii) disposing of all nesting material at the end of each season. We test the hypothesis that management will reduce the transmission of BFDV to nestlings by using a reciprocal repeated measures experimental design implemented *in situ*. We also test whether management improves nestling body condition and fledging success.

## Results

### Viral prevalence and load

**Figure 1:**
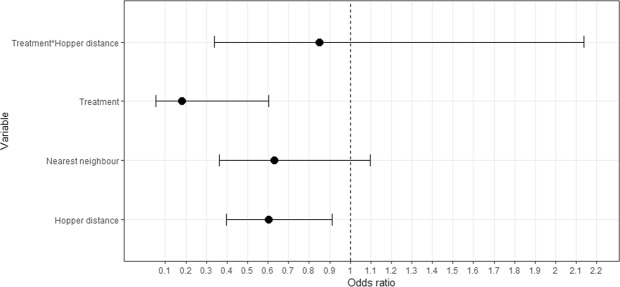
The association of treatment, distance to nearest feeding hopper, distance to nearest neighbouring nest site and the interaction between treatment and distance to nearest feeding hopper with the probability of BFDV infection in 45-day old Mauritius parakeet nestlings produced over the three experimental breeding seasons. Variable specific odds ratios are denoted by the filled circles along with their associated 95% CIs.

**Figure 2:**
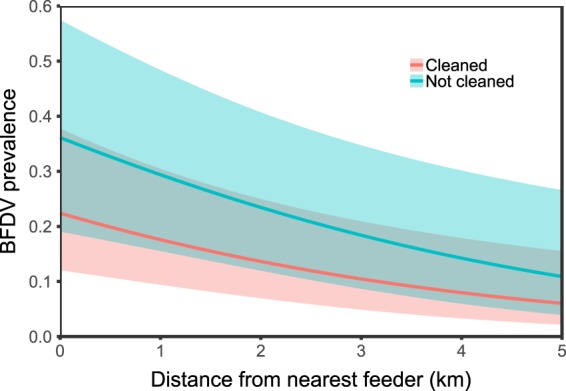
Predicted probability of Mauritius parakeet nestlings becoming infected with BFDV as a result of nest site treatment with increasing distance from the nearest feeding station, with female parent and breeding season specified as random intercept effects. Shaded areas are 95% prediction intervals.

For the binomial probability of infection in nestlings we found two equally supported candidate models that included the additive effects of treatment, distance to nearest feeding hopper, the interaction between these two factors, as well as the additive effect of distance to the nearest neighbour (Table 1a, Fig. 1). When the interaction between treatment and distance to nearest feeding hopper was explored, it indicated that the probability of nestling infection with BFDV was lower both when the distance to supplementary feeding hopper was greater and when nest site management is done (Fig. 2). Prevalence of BFDV-infected nestlings across years and with current BFDV nest site management was 13.9% (SE+/− 5.31%) and our experimental models estimated this to be, on average, 11% lower than if no management was applied. However, we found no strong links between management actions and individual nestling viral load, with the null model as most parsimonious (Table 1b, Supplementary Table S1b). So, whilst management reduced the proportion of nestlings infected with BFDV it had no apparent impact on individual infection intensity.

**Table 1:** A comparison of the ten generalised linear models analysing (a) the predicted probability of BFDV infection in 45-day old Mauritius parakeet nestlings over seven breeding seasons (2009/10 to 2015/16), and (b) individual BFDV load in 45-day old Mauritius parakeet nestlings over the three experimental breeding seasons (2013/14 to 2015/16).

Rank	Model	K	AIC_c_	ΔAIC_c_	AIC_c_ weights
**(a)**					
1	T + SF + NN	6	764.25	0.00	0.66
2	T + SF + NN + T*SF	7	766.18	1.94	0.25
3	T + NN	5	769.43	5.19	0.05
4	T + SF	5	771.14	6.89	0.02
5	SF + NN	5	771.95	7.70	0.01
6	SF + T + T*SF	6	773.16	8.91	0.01
7	NN	4	775.27	11.03	0.00
8	SF	4	778.97	14.73	0.00
9	T	4	779.83	15.58	0.00
10	Null model	3	785.42	21.17	0.00
**(b)**					
1	Null model	4	−1817.35	0.00	0.98
2	SF	5	−1808.64	8.72	0.01
3	T	5	−1806.27	11.08	0.00
4	NN	5	−1806.07	11.28	0.00
5	T + SF	6	−1797.50	19.86	0.00
6	SF + NN	6	−1796.81	20.54	0.00
7	T + NN	6	−1794.96	22.40	0.00
8	SF + T + T*SF	7	−1786.76	30.59	0.00
9	T + SF + NN	7	−1785.65	31.70	0.00
10	T + SF + NN + T*SF	8	−1774.94	42.42	0.00

Management factors related to BFDV prevalence include treatment (T), distance to the nearest supplementary feeding station (SF) and distance to nearest neighbouring nest site (NN) based on Akaike’s information criterion corrected for finite sample size (AIC_c_) and weights (AIC_c_ weights). All models were run with the nesting female and breeding season as random intercept effects. K denotes the number of parameters in each model and models are ranked according to their ΔAIC_c_.

### Nestling fitness impacts

**Figure 3:**
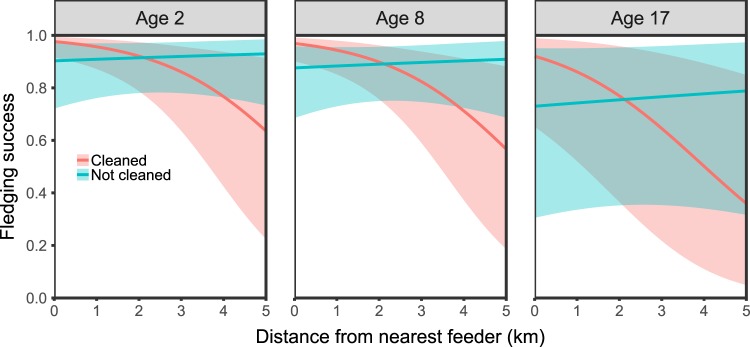
Predicted probability of Mauritius parakeet nestlings fledging as a function of nest site treatment and increasing distance from supplementary feeding hoppers. Panels indicate predicted probabilities over the experimental breeding seasons in breeding females across three discrete age cohorts (5, 7 and 11 years corresponding approximately to the 25^th^, 50^th^ and 75^th^ quantile of the distribution of age of birds in our dataset), with female parent and breeding season specified as random intercept effects. Shaded areas are 95% prediction intervals.

Fledge success was determined by the additive effects of treatment, distance to nearest feeding hopper and dam age (two equally supported models; Table 2). Counter to expectations there was a greater proportion of chicks fledged from control nests (i.e. those not managed with BFDV biosecurity; 83% vs. 79%), although only the interaction between treatment and the distance to nearest feeding hopper was found to be a significant predictor of the probability of fledging (Odds ratio = 0.49, 95% CI 0.29–0.80; Fig. 3, Supplementary Table S1c). Whereas there was a clear decline in the probability of fledge success with distance away from feeding hoppers in managed nests this was not apparent in control nests. This pattern was found to be consistent across age cohorts, but with older females experiencing a steeper decline with increasing distance from feeding hopper in treatment sites, and an overall lower probability of fledge success than younger females (Fig. 3). Only a single model determined fledgling body condition. This model included all of the assessed variables (Table 3), none of which were found to be predictive of nestling body condition (95% CIs overlap 0, Supplementary Table S1d).

**Table 2:** A comparison of the ten generalised linear models analysing the probability of fledge success of Mauritius parakeet nestlings over the three experimental breeding seasons (2013/14 to 2015/16).

Rank	Model	K	AIC_c_	ΔAIC_c_	AIC_c_ weights
1	SF + T + F + F2 + T*SF	8	446.90	0.00	0.42
2	SF + T + T*SF	6	448.22	1.32	0.22
3	F + F2	5	450.27	3.37	0.08
4	SF + F + F2	6	450.28	3.38	0.08
5	Null model	3	451.28	4.38	0.05
6	T + F + F2	6	451.46	4.56	0.04
7	SF	4	451.55	4.64	0.04
8	SF + T + F + F2	7	451.90	5.00	0.03
9	T	4	452.48	5.57	0.03
10	SF + T	5	453.12	6.22	0.02

Factors related to fledge success include treatment (T), distance to the nearest supplementary feeding station (SF) and the linear (F) and quadratic terms (F2) of dam age based on Akaike’s information criterion corrected for finite sample size (AIC_c_) and weights (AIC_c_ weights). All models were run with the nesting female and breeding season as random intercept effects. K denotes the number of parameters in each model and models are ranked according to their ΔAIC_c_.

**Table 3:** A comparison of the 16 generalised linear models analysing body condition (^mass^/_wing length_) of Mauritius parakeet nestlings over the three experimental breeding seasons (2013/14 to 2015/16).

Rank	Model	K	AIC_c_	ΔAIC_c_	AIC_c_ weights
1	T + VL + F + F2 + SF + T*SF	10	4672.95	0.00	0.79
2	T + VL + F + F2 + SF	9	4676.98	4.03	0.11
3	T + VL + F + F2	8	4678.43	5.47	0.05
4	VL + F + F2 + SF	7	4679.41	6.46	0.03
5	VL + F + F2	7	4680.71	7.76	0.02
6	T + F + F2 + SF + T*SF	9	4778.75	105.80	0.00
7	T + F + F2 + SF	8	4782.68	109.73	0.00
8	T + F + F2	8	4803.71	130.76	0.00
9	F + F2	6	4821.25	148.29	0.00
10	T + VL + SF + T*SF	8	4935.54	262.59	0.00
11	T + VL + SF	7	4939.14	266.19	0.00
12	T + VL	6	4939.79	266.83	0.00
13	VL	5	4942.37	269.42	0.00
14	T	5	5062.80	389.85	0.00
15	SF	5	5064.72	391.77	0.00
16	Null model	4	5082.66	409.71	0.00

Factors related to body condition include treatment (T), distance to the nearest supplementary feeding station (SF), individual BFDV load (VL) and the linear (F) and quadratic terms (F2) of dam age based on Akaike’s information criterion corrected for finite sample size (AIC_c_) and weights (AIC_c_ weights). All models were run with the nesting female and breeding season as random intercept effects. K denotes the number of parameters in each model and models are ranked according to their ΔAIC_c_.

## Discussion

Our results illustrate the complexity of applying disease management strategies in the context of endangered species conservation, and the vital importance of critically evaluating the effectiveness of actions. We found evidence that nest site management led to a small reduction in the probability of a brood becoming infected with BFDV, although the same management was not found to affect BFDV load or the body condition of chicks. Conversely, we found that nest management does not enhance Mauritius parakeet recovery and may even hinder it (albeit by a small amount on otherwise high fecundity). Our experiment does not provide an explanation for the lower fecundity in managed nests, but we suggest two possibilities; that the chemical treatments, as used, may negatively affect parakeet eggs and nestlings or, perhaps, that the longer processing times required with the biosecurity protocols add to nest disturbance. Indeed, both Virkon specifically and quaternary ammonia-based disinfectants have been shown to impact on shell porosity when applied directly to eggs, thus reducing their hatchability^34,35^. Given our results, we recommend a change to current management, possibly beginning with an experimental reduction in the number of nests managed, or with a shortening of biosecurity protocols to reduce potential stress. However, since the results are relative to the conditions of our study, we also caution against a general interpretation that biosecurity is not important. Rather we suggest that the current method is not achieving its intended purpose.

BFDV prevalence was also driven partly by the proximity of nests to feeding hoppers. Parents nesting closer to feeding hoppers and aggregating around them may be facilitating BFDV transmission through increased contact rates^36^. Supplementary feeding stations are known to facilitate pathogen transmission across a broad range of host species globally and their use should be carefully managed to ensure they are beneficial in species recovery^37,38^.

The value of assessing EID management options through experimental evaluation is also illustrated by a handful of recent attempts at *in situ* management of amphibian chytridiomycoses. For example, despite the initial success of trials to reduce mortality through repeated anti-fungal treatment of *Batrachochytrium dendrobatidis* infection in the mountain chicken frog (*Leptodactylus fallax*), these benefits were lost on cessation of treatment^9^. Whilst the means objective of clearing infection was temporarily met, from the broader conservation perspective the fundamental objective of population recovery was unachievable in the long term. Conversely, in a simplified system with a single host and the ability to also treat the surrounding environment, experimental evaluation showed the beneficial outcomes of *B. dendrobatidis* management might be sustainable in Mallorcan midwife toads (*Alytes muletensis*)^10^. When considering management options for *Batrachochytrium salamandrivorans* in fire salamanders (*Salamandra salamandra*), models showed that even treatment actions that led to considerable increases in survival or reductions in transmission were unlikely to be effective in the long term and, in fact, prolonging survival of infected individuals may instead encourage pathogen transmission and worsen population-level impacts^39^. Our experimental results and these examples clearly illustrate two important messages related to the management of EIDs in wildlife conservation.

Firstly, in the crisis scenarios commonly faced by critically endangered species, initial decisions about disease risk management inevitably draw on available knowledge and expert opinion from wildlife health professionals^40^. Advisory panels often combine very different experiences (such as zoo veterinarians and field rangers), and actions may be extrapolated from different contexts (e.g. *ex-situ* treatments applied in the wild)^41^. For example, in Mauritius parakeets, the initial decision of applying biosecurity and feeding was made under the assumptions that treatments known to reduce infection would be beneficial for population persistence. Given the critical status of the species and the potentially severe threat posed by BFDV, the initial decision to apply disinfection protocols was urgently required and therefore necessarily conservative.

Although such limitations are a necessity when initiating recovery programs, decisions can be re-evaluated critically by monitoring the outcomes of implemented actions^42^, yet such re-evaluations are surprisingly rare^9,10,39^. Not measuring the efficacy of actions thought to reduce transmission of EIDs reflects a general pattern of poor integration of strategic monitoring in management^43,44^; something that frequently leads to suboptimal conservation and the development of conservation dogmas^45^. In our study system, the evaluation of nest management provided by this study has led us to reconsider whether to continue the intensive biosecurity protocols, which we had assumed were necessary for Mauritius parakeet persistence.

Secondly, monitoring the effectiveness of management must maintain focus on the fundamental objective of that management. In our case, management aimed to reduce the transmission of BFDV. However, BFDV in itself was considered important because of its potential negative effects on the fundamental management objective, the recovery of the threatened host species. In this sense, reducing the prevalence and load of BFDV represents a means objective to species recovery, but one that is surrounded by substantial uncertainty in the way BFDV is transmitted, the risks it poses to the Mauritius parakeets and our ability to manage it. Our experiment suggested nest management could provide a small (on average 11%) reduction in the probability of infection of a brood with BFDV. If the evaluation focused exclusively on the target of BFDV prevalence, nest management may thus appear desirable. However, this marginal benefit might be offset by the tendency of managed nests to have lower fledging success (a component vital rate of population growth). Such a trade-off clearly illustrates why incorrectly focusing monitoring on means objectives can increase the risk of suboptimal conservation outcomes^43^.

Both poor monitoring of management outcomes and a tendency to focus on means objectives can be addressed through a better placement of science within management decision making. The emergence of BFDV in numerous wild populations has led to a substantial contribution of interesting and valuable research^26,46,47^, yet managers remain uncertain on how best to respond. Our experiment was a direct response to manager requests to critically review long-running and increasingly demanding nest site management (over 13 years with a population that increased in size from 39 known breeding pairs in 2004 to 102 pairs by the start of our experiment^48^). We have not explicitly considered the logistic and financial cost of management but, as this is a substantial burden on the recovery program, it should be rewarded with improved conservation outcomes. Rather than simply measure BFDV, we also distinguished the means and fundamental objectives driving management of this EID. Structuring conservation science within management decision making ensures research findings are not only interesting, but relevant.

Faced with an increasing frequency of EIDs, managers need to make hard decisions about whether to alter management to reduce their spread or impact. Frustratingly, in the crisis scenarios that many endangered species face, these choices often need to be made quickly and in the face of substantial uncertainty. Given the high risks to populations or species from making the wrong choice (e.g. extinction) it is essential to evaluate whether management is achieving predicted outcomes. Targeted monitoring and, where possible, manipulation of the focal systems provides a powerful framework to advance threatened species conservation. When making these choices managers should carefully compare consequences against fundamentally important objectives, usually linked to the recovery of the host species.

## Methods

### PBFD and the transmission of BFDV

PBFD is typically characterized by chronic symmetrical feather abnormalities and dystrophy but can also induce severe claw and beak deformities^49–51^ and its immunosuppressant nature increases host susceptibility to secondary infection^23,52^. BFDV, a member of the Circoviridae family^53^, is considered to demonstrate high environmental persistence owing to its ability to infect a broad range of closely related host species^26^ and is transmissible both horizontally (through contact with contaminated feather dust, surfaces or objects^52^), and vertically (from a female to her offspring^23,27^). Whilst PBFD can be fatal and most commonly affects birds up to three years of age^23^, infected individuals can recover from acute presentation of the disease^54^. Other individuals may not display any clinical signs of infection despite carrying the virus^23^. BFDV within Mauritius parakeet nestlings has been continuously monitored by taking blood samples from all 45-day old nestlings produced annually since 2005^33,36^. In addition to collection of a blood sample in the field, each nestling is given a unique combination of leg bands, is assigned a Studbook ID and has morphometric data collected; including body mass, wing length and tail length.

### Experimental design

**Figure 4:**
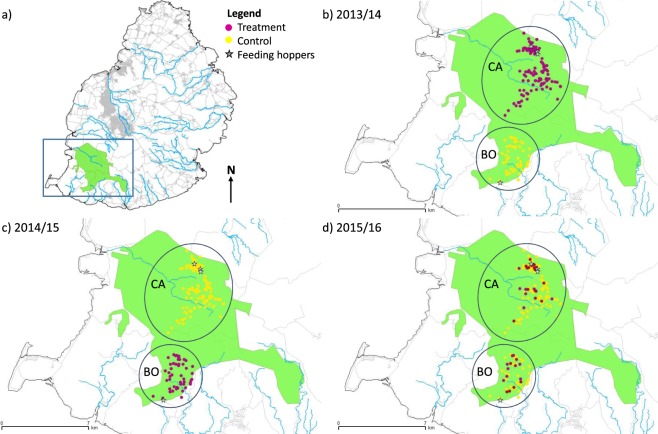
(**a**) The location of the remaining Mauritius parakeet breeding populations in the Black River Gorges National Park in the south-west of Mauritius, (**b**) the 2013/14 breeding season experimental design, (**c**) the 2014/15 breeding season reciprocal experimental design, and (**d**) the 2015/16 breeding season mixed experimental design. CA = Camp, BO = Bel Ombre.

Two experimental groups were allocated based on natural geographic separation of the population into two sub-populations (Bel Ombre in the South and Camp in the North, Fig. 4). There is little evidence of natural parakeet dispersal between these subpopulations^33^ despite regular artificial movements during cross-fostering, captive breeding and release management prior to the initial outbreak of PBFD in 2005. Both sub-populations are found in similar forested and protected habitat within the Black River Gorges National Park and are assumed to face similar climatic conditions, as they are separated by only about 1.8 km. A key difference, however, is that the number of birds is much greater within the northern Camp group (87 vs. 39 known active natural and artificial nest sites in the 2015/16 breeding season), probably due to the longer and more intense management focus that area has received.

We implemented an experiment over three breeding seasons (2013/14, 2014/15 and 2015/16). This experiment was conducted under the University of Kent ethical guidelines (0018-DF-16) with veterinary consultation and supervision by A. Greenwood, and approved by both the Mauritian Wildlife Foundation and the Mauritius National Parks and Conservation Services. In breeding season one we undertook standard PBFD management in Camp (n = 73 nest sites), involving wearing medical barrier suits whilst accessing the nests, disposing of all old nesting material and disinfecting these nest boxes with a hospital-grade disinfectant selected due to its virucidal efficacy^55,56^ (Virex, comprising a quaternary ammonium chloride base or Virkon, comprising a potassium peroxymonosulfate base, depending on availability) prior to the breeding season. No management measures were applied in Bel Ombre (n = 29 nest sites; Fig. 4b). In breeding season two these treatments were swapped in a reciprocal design so that PBFD management was undertaken in Bel Ombre (n = 33 nest sites) but not in Camp (n = 74 nest sites; Fig. 4c). In the final breeding season, 31 nest sites (25% of all active sites) across both sub-populations were selected for treatment to account for any variation between these two groups (Fig. 4d). In this experiment our treatment refers to where PBFD management is used compared to our control where PBFD management is not. Across both groups all other management actions, including supplementary feeding, remained as normal^48,57,58^.

### Laboratory analysis

Two methods were used in the lab to provide both a viral prevalence dataset as well as an assessment of individual nestling viral load from the nestling blood samples collected. Host and viral DNA, where present, were extracted from 50 to 100 μl of host whole blood using a combination of DIGSOL extraction buffer and 10 mg/mL proteinase K^59^. Extractions were quantified using a Qubit dsDNA Assay Kit and standardised to approximately 25 ng/μl prior to screening for BFDV through standard PCR, and to 10 ng/μl for quantification using real-time PCR (rtPCR).

Standard PCR protocols used to detect BFDV infection status of an individual were as detailed in Kundu *et al*. (2012). In brief, the PCR assay targeted a 717-bp region of the r*eplicase* gene^60^ and comprised 1 μl of extracted host DNA template, 5 μl MyTaq^TM^ HS Red Mix (Bioline), 0.2 μl each of the forward and reverse primers at 10 pmol/μl and was made up to 10 μl with double-distilled water. PCR annealing temperature was adjusted to 60 °C, as per manufacturer’s guidelines, for 30 cycles and products were visualized on a 1.5% agarose gel. Both a known BFDV positive Mauritius parakeet sample and a negative control were included in each PCR batch.

For rtPCR protocols an assay also targeting the *replicase* gene was used to quantify individual viral load^61^, with each reaction consisted of 10 μl iTaq Universal Probes Supermix (Bio-Rad Inc.), 0.8 μl of each of the forward (5′-TGGGTGGCTACCTTATTG-3′) and reverse (5′-GGCTTATTGCTCGTGATAA-3′) primers, 0.2 μl of a FAM-labelled fluorescent probe (5′FAM-CTCTGCGACCGTTACCCACA-3′TAM), 5 μl of DNA template and made up to 20 μl with double-distilled water. Cycle conditions were as follows: initial denaturation of 5 min at 95 °C; followed by 40 cycles of: 5 s at 95 °C and 30 s at 60 °C. All 96-well plates included two positive controls from a high viral load Mauritius parakeet individual (amplification at ~10 cycles) for the purposes of standardisation between runs and two negative controls to ensure no contamination was present. Each individual was run in duplicate. If the repeats did not amplify within one PCR cycle of one another, a third replicate was performed. The averaged C_T_ values for each individual were then converted into a relative estimate of viral load^62^ using the equation: Viral load = 2^(−ΔCT)^

### Data analysis

#### Viral prevalence

Using the data generated from standard PCR, generalised linear mixed models (GLMMs) were run with the lme4^63^ package in R version 3.4.3^64^ using a binomial response variable accounting for the number of BFDV-positive and -negative nestlings per nest site, and setting a binomial error distribution and a logit link function^29^. To thoroughly investigate efficacy of management we also included the long-term data on nestling infection with BFDV systematically collected across both sub-populations between 2009 and 2013, where BFDV management was always applied (Supplementary Table S2). We evaluated a set of candidate models investigating the effects of three management related factors on the proportion of BFDV infected nestlings per brood (binomial response variable given by number of BFDV-positive nestlings to the number of negative nestlings tested): distance to the nearest feeding hopper (km), distance to the nearest neighbouring nest site (km) and our experimental treatment. Female parent and breeding season were used as random intercept effects to account for both the vertical and horizontal viral transmission pathways (as females generally nest at the same site year on year) and for any abiotic variation between breeding seasons. We were aware that each sub-population had a different placement of feeding hoppers relative to nests sites, resulting in differences in the likelihood that breeders would use them (Camp, mean distance nest to feeding hopper = 0.76 ± 0.08 km (SE); Bel Ombre, mean distance nest to feeding hopper = 2.38 ± 0.14 km (SE); t(240) = 18.06, p < 0.001; Fig. 4)^36^. Given the difference in proximity to feeding hoppers between sub-populations and previous indications that feeding hoppers are another potential site of human-influenced BFDV transmission, we included an interaction between treatment and distance to nearest feeding hopper in the candidate model set. Sub-population, controlled for in the experimental design, was inherently linked with year and treatment so was therefore not included as a factor in the model set. We selected the most parsimonious model based on the lowest Akaike’s information criterion corrected for finite sample size (AIC_C_). As more than one model was within 2 delta AIC_c_, and therefore equally plausible, we used model averaging (*AICcmodavg* package^65^) to estimate predicted parameter values.

#### Individual viral load

For the assessment of individual viral load derived from the qPCR data, GLMMs were run using the same response variables as for the viral prevalence dataset and spanned the three experimental breeding seasons from 2013 to 2016. Viral load values were logged and a Gaussian distribution was used, including both female parent and breeding season as random intercept effects. We selected the most parsimonious model based on the lowest AIC_c_.

#### Nestling fitness impacts

GLMMs were run on two parameters to assess potential population impacts of biosecurity protocols on productivity and individual fitness across the three experimental breeding seasons (Supplementary Table S3). The first set of candidate models evaluated the effects of distance to nearest feeding hopper (km), treatment and both the linear and quadratic terms for dam age on the proportion of nestlings fledged (n = 311 nest sites), using a Gaussian distribution and with female parent and breeding season used as random intercept effects. Viral load was not assessed as a factor to avoid bias in results due to the deficit of data from nestlings that didn’t survive to the point of sampling. We developed a second set of candidate models to assess the impacts of distance to nearest feeding hopper (km), treatment, both the linear and quadratic terms for dam age and logged viral load on body mass (g) (n = 559 fledglings), with wing length (cm) used to correct for body size^66^. Female parent and breeding season were used as random intercept effects to account for variability across broods due to abiotic or genetic factors.

## Supplementary information

Fogell et al. Supplementary Material

## Data Availability

This statement confirms that, should the manuscript be accepted, then data supporting the results will be archived in the Kent Academic Repository.
