# Supplementary material for: Hygiene and biosecurity protocols reduce infection prevalence but do not improve fledging success in an endangered parrot
Source: Sci Rep. 2019 Mar 18;9:4779. doi: 10.1038/s41598-019-41323-w (PMC6423005; doi:10.1038/s41598-019-41323-w)
Supplement: Supplementary file 1 — Fogell et al. Supplementary Material [file 41598_2019_41323_MOESM1_ESM.pdf]

TITLE: Hygiene and biosecurity protocols reduce infection prevalence but do not improve fledging success in an endangered parrot

Deborah J. Fogell[1,2*], Jim J. Groombridge[1], Simon Tollington[1,3], Stefano Canessa[4], Sion Henshaw[5], Nicolas Zuel[5], Carl G. Jones[5, 6], Andrew Greenwood[7] and John G. Ewen[2]

Supplementary Table S1 A summary of model averaged coefficients and effect sizes for the generalised linear mixed effect candidate models analysing a.) the probability of BFDV infection in 45-day old Mauritius parakeet nestlings over seven breeding seasons (2009/10 to 2015/16), b.) individual BFDV load, c.) probability of fledge success, and d.) body condition ($^{mass}/_{wing\ length}$) of Mauritius parakeet nestlings over the three experimental breeding seasons (2013/14 to 2015/16).

Factor	Model Estimate	95% Confidence Interval
**a.**		
Treatment	-0.74	-1.26 – -0.22
Nearest hopper	-0.22	-0.40 – -0.04
Nearest neighbour	-0.20	-0.44 – 0.04
Treatment*Nearest hopper	-0.07	-0.47 – 0.33
**b.**		
Treatment	0	-0.01 – 0.01
Nearest hopper	0	-0.01 – 0
Nearest neighbour	0	-0.01 – 0
Treatment*Nearest hopper	0	-0.01 – 0
**c.**		
Treatment	1.3	-0.04 – 2.63
Nearest hopper	0.02	-0.39 – 0.42
Treatment*Nearest hopper	-0.71	-1.2 – -0.22
Dam Age	1.12	-0.48 – 2.73
(Dam Age)^2	-1.47	-3.08 – 0.15
**d.**		
Treatment	-1.95	-7.79 – 3.89
Nearest hopper	-2.86	-6.46 – 0.75
Treatment*Nearest hopper	4.36	-1.24 – 9.96
Log(Viral Load)	0.18	-2.59 – 2.95
Dam Age	5.92	-8.31 – 20.14
(Dam Age)^2	-7.64	-21.75 – 6.46

Supplementary Table S2 A summary of the brood-level prevalence of BFDV in 45-day old Mauritius parakeet nestlings over seven breeding seasons (2009/10 to 2015/16) and individual Mauritius parakeet nestling BFDV load over the three experimental breeding seasons (2013/14 to 2015/16) broken down by subpopulation and treatment.

**Breeding season**	**Total nestlings screened**	**Total nest sites**	**Mean brood prevalence**		**Nestling viral load (Min – Max)**	
			**Treated**	**Untreated**	**Treated**	**Untreated**
Bel Ombre						
2009/10	27	13	0.10	*NA*	*NA*	*NA*
2010/11	24	14	0.35	*NA*	*NA*	*NA*
2011/12	19	12	0.28	*NA*	*NA*	*NA*
2012/13	46	23	0.00	*NA*	*NA*	*NA*
2013/14	53	23	*NA*	0.45	*NA*	1.03E-05 (7.05E-08 – 2.22E-04)
2014/15	58	28	0.03	*NA*	2.03E-03 (0.00E+00 – 1.19E-01)	*NA*
2015/16	73	35	0.00	0.00	6.48E-09 (0.00E+00 – 1.30E-07)	4.83E-09 (0.00E+00 – 1.05E-07)
Camp						
2009/10	104	48	0.30	*NA*	*NA*	*NA*
2010/11	98	48	0.40	*NA*	*NA*	*NA*
2011/12	97	49	0.25	*NA*	*NA*	*NA*
2012/13	102	54	0.05	*NA*	*NA*	*NA*
2013/14	101	46	0.39	*NA*	5.84E-02 (0.00E+00 – 2.07E+00)	*NA*
2014/15	141	63	*NA*	0.28	*NA*	3.00E-02 (0.00E+00 – 3.23E+00)
2015/16	131	64	0.04	0.08	3.49E-03 (0.00E+00 – 1.29E-01)	4.67E-02 (0.00E+00 – 2.24E+00)

Supplementary Table S3 A summary of the body condition ($^{mass}/_{wing\ length}$) and fledge success of Mauritius parakeet nestlings over the three experimental breeding seasons (2013/14 to 2015/16) broken down by treatment.

Breeding season	Body condition (Min – Max)		Fledge success	
	Treated	Untreated	Treated	Untreated
2013/14	1.32 (0.90 – 1.87)	1.24 (0.88 – 1.85)	0.67	0.81
2014/15	1.33 (0.92 – 3.14)	1.17 (0.86 – 1.83)	0.92	0.83
2015/16	1.38 (0.90 – 3.27)	1.31 (0.93 – 2.15)	0.97	0.83
